# Dynamic stretching is not detrimental to neuromechanical and sensorimotor performance of ankle plantarflexors

**DOI:** 10.1111/sms.13321

**Published:** 2018-11-08

**Authors:** George M. Pamboris, Marika Noorkoiv, Vasilios Baltzopoulos, Amir A. Mohagheghi

**Affiliations:** ^1^ Centre for Human Performance, Exercise and Rehabilitation Brunel University London Uxbridge UK; ^2^ Institute of Environment, Health and Societies Brunel University London Uxbridge UK; ^3^ Research Institute for Sport and Exercise Sciences (RISES) Liverpool John Moores University Liverpool UK; ^4^ University of Social Welfare and Rehabilitation Sciences Tehran Iran

**Keywords:** dynamometry, muscle strain, range of motion, tendon strain, ultrasonography

## Abstract

The acute effects of two dynamic stretching (DS) protocols on changes in the ankle range of motion (RoM), neuromechanical, and sensorimotor properties of the plantarflexor muscle group were examined. Eighteen participants received slow (SDS) or fast dynamic stretching (FDS) on two separate days. Outcome measures were assessed pre‐ and 2 minutes post‐interventions, and included maximum dorsiflexion angle, maximum isometric torque at neutral ankle position, maximum concentric and eccentric torques, force matching capacity, joint position sense and medial gastrocnemius muscle and tendon strain. Possibly and likely small increases in dorsiflexion RoM were observed after SDS (mean ± 90% confidence intervals; 1.8 ± 1.2°) and FDS (2.1 ± 1.2°), respectively. Very likely moderate decreases in muscle strain after SDS (−38.0 ± 20.6%) and possibly small decrease after FDS (−13.6 ± 21.2%) were observed. SDS resulted in a likely beneficial small increase in tendon strain (25.3 ± 29.7%) and a likely beneficial moderate increase after FDS (41.4 ± 44.9%). Effects on strength were inconsistent. Possibly small effect on positional error after SDS (−27.1 ± 37.5%), but no clear effect after FDS was observed. Both DS protocols increased RoM, and this was more due to an increase in tendon elongation rather than the muscle. However, SDS showed greater improvement than FDS in both neuromechanical and sensorimotor performance, and hence, SDS can be recommended as part of warm‐up in sporting contexts.

## 1 INTRODUCTION

Static stretching (SS) has been recently criticized for impairing muscular performance reflected, for example, in maximal voluntary strength, muscle power, sprint time, and jump height.^1^ This has resulted in a shift from SS back to dynamic stretching (DS), recommending that DS may be included in the stretching component of warm‐ups to increase task‐specific range of motion (RoM), and facilitate stretch‐shortening cycle soon before an activity, and/or when a full pre‐activity routine is not completed.^1^ Supporting this view, previous studies have shown that there is no stretch‐induced strength loss after DS^2^ and that DS may improve isometric and isotonic contractions.^3^, ^4^ Recent evidence also indicates that DS could facilitate power production^3^, ^4^ and improve sprint time^5^ and jump height.^6^

However, the mechanisms that seek to explain the advantage of DS are only suggestive and include increased heart rate, elevation of core and muscle temperature,^3^, ^7^, ^8^ and increased transmission rate and metabolism.^7^ Specific rehearsal of movement patterns that may enhance proprioception,^8^ and an increase in neuromuscular activity^9^, ^10^ that is possibly linked to post‐activation potentiation (PAP)^3^ may also lead to strength and power enhancement.

One possible effect of an elevated muscle temperature resulting from DS is a decrease in its viscosity^7^ resulting in a decrease of passive torque at end RoM and increase in joint RoM.^11^, ^12^ Thus, one would expect a change in muscle‐tendon unit (MTU) mechanical properties as a result of DS. Herda et al^13^ used dynamometry to examine the effects of DS on passive MTU stiffness as a possible mechanism of increased RoM of the target muscle by showing the relationship between the increased joint angle and decreased passive torque developed as resistance to motion. Samukawa et al^14^ using B‐mode ultrasonography observed an increase in ankle dorsiflexion and a proximal displacement of the muscle‐tendon junction of the medial head of the gastrocnemius during standing after DS, suggesting that an increase in tendon length contributes to the increased ankle RoM. Such changes in the mechanical properties of the muscle, tendon, or MTU can affect MTU functional properties by possibly shifting the working range in the force‐length and force‐velocity relationships.^15^ Contrasting views on the potential effect of DS on MTU properties are, however, reported. Mizuno and Umemura^16^ reported that a DS technique did not change the mechanical properties of the MTU, attributing the change in RoM to enhance stretch tolerance.

Dynamic stretching protocols used in the studies above were varied in their respective methodology greatly. For example, one involved contracting the muscle group “antagonist” to the stretched target muscle group,^16^ while the others involved contracting the muscle group “agonist” to the stretched target muscle group.^13^, ^14^ Discrepancies among the results of studies which employed DS necessitate further examination of this commonly employed warm‐up intervention given the different neuromuscular activation effects of the above contraction modalities. In particular, the effects of a DS protocol consisting of contraction of the “agonist” muscle group on the passive mechanical properties of the MTU which may influence maximal voluntary strength of the target muscles are unclear. Additionally, the appropriate pre‐participation DS protocol (eg, number and rate of repetitions, intensity of muscle stretching, contracting muscle group) for affecting maximal strength in sports activities has not been determined. For plantarflexors, it has been demonstrated that a faster DS (100 beats/min) induced greater increase in jump height than a slower one (50 beats/min),^17^ however, the mechanism underlying this effect was not explained. Thus, limited data exist which can describe the mechanisms for the enhancement of RoM after DS, and it is not known whether changes in the stretch tolerance are similarly influential in DS as they are in SS and proprioceptive neuromuscular facilitation (PNF) stretching.

Other performance related parameters could also be affected by DS. Proprioceptive acuity defined as an individual's ability to sense joint position, movement, and force as a means to discriminate body movement^18^ is one such parameter, but available research on the potential of DS to compromise proprioceptive acuity is limited. It is suggested that changing the mechanical properties of the MTU may directly impair its force generating capacity,^19^ influence neural activation patterns^19^ and sensorimotor performance (such as ability to scale volitional force and joint position sense). These may be measured as the force matching error in the reproduction of a target force by the involved musculature to a given percentage of the volitional peak force and the replication of a target joint position.^20^

In this context, the aim of this study was to investigate the potential effect of DS on performance via examining its effect on the neuromuscular and sensorimotor mechanisms. In addition to muscle length, we assessed ankle joint proprioceptive acuity, force matching error, and changes to muscle behavior (contributing to joint RoM) before and after DS.

## 2 MATERIALS AND METHODS

### 2.1 Participants

Eighteen active participants (nine Males: age 28 ± 3 years; height: 1.80 ± 0.07 m; body mass: 78 ± 8 kg; nine Females: age 29 ± 6 years; height: 1.65 ± 0.06 m; body mass: 63 ± 10 kg) with no history of lower limb injury within 6 months prior to the study volunteered for this study. All participants completed a medical questionnaire and provided written informed consent. We instructed the participants to refrain from vigorous physical activity for 48 hours before the testing sessions in order to avoid any potential carryover effects and promote neuromuscular recovery. Ethics approval was granted by the Research Ethics Committee of the Department of Life Sciences at Brunel University London, and the study was completed in accordance with the Declaration of Helsinki.

As the fluctuation in female steroid hormones during the menstrual cycle does not have substantial influence on the mechanical properties of the human muscle and tendon in vivo,^21^ and the examination of any potential effects of the menstrual cycle was beyond the scope of the current study, women with a regular menstrual cycle lasting between 28 and 32 days were included and tested at a non‐specific period. Additionally, no significant effect of sex has been reported on stretching‐induced changes in muscle‐tendon unit stiffness and RoM,^22^ so both genders were included in the study.

### 2.2 Experimental design

**Figure 1:**
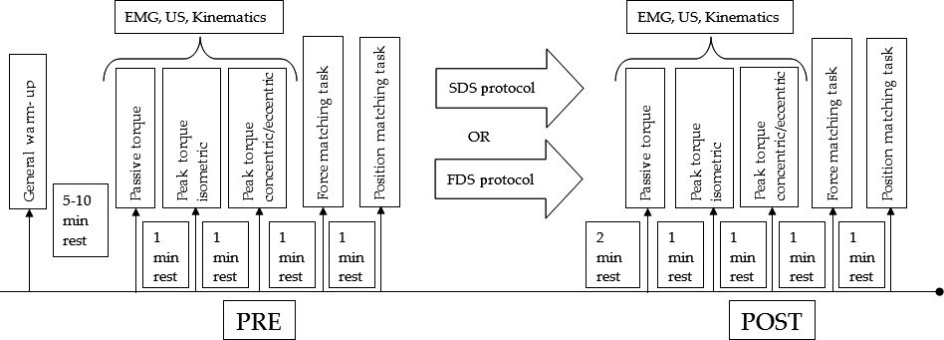
Schematic representation of the study protocol. EMG, Electromyography; FDS, Fast Dynamic Stretching; SDS, Slow Dynamic Stretching; US, Ultrasound

The experimental setup is shown in Figure 1. We conducted a randomized crossover‐controlled trial where participants visited the laboratory on two occasions separated by at least 48 hours. We provided a full familiarization of the testing procedure on both visits before data were collected. On the first visit, the participants underwent a warm‐up and either a fast DS protocol (FDS – 100 beats/min) or a slow DS protocol (SDS – 50 beats/min). SDS and FDS were performed in random order. We used a blind method where the participant did not know the recorded scores, to minimize internal validity bias. Outcome measures were taken at baseline and remeasured 2‐minute post‐DS protocols. The 2‐minute interval was similar to the minimum period between warm‐up and start of a game/training session as used by previous researchers.^5^ The reliability of the employed measurement techniques has already been reported in the literature.^23^, ^24^, ^25^

### 2.3 Instrumentation

**Figure 2:**
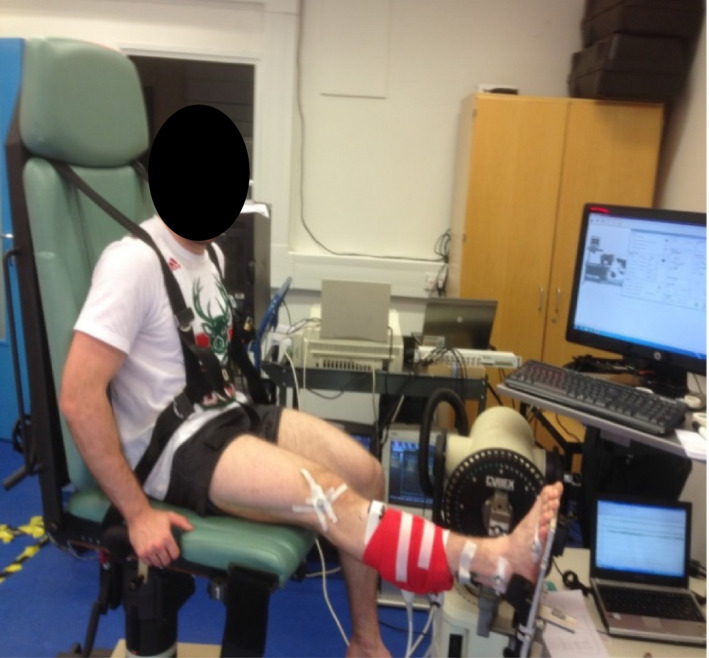
Participant on the dynamometer for the assessment of ankle plantarflexors neuromuscular performance

An isokinetic dynamometer (Cybex NORM, New York, USA) was used to assess (a) passive torque about the ankle joint during dorsiflexion, (b) peak plantarflexion torque during isometric and isokinetic modes of contractions, (c) passive joint position sense, and (d) force matching error. The position of the participant during testing is illustrated in Figure 2. The participants sat upright (hip angle at ~85°) in the chair with the knee fully extended (0°), and the ankle at neutral position (0°) with the sole of the foot perpendicular to the shank, and the midline between the lateral and medial malleoli aligned with the center of rotation of the dynamometer. To isolate ankle movement, stabilizing straps (not shown) were firmly tightened over the foot, thigh, and chest, to minimize heel displacement from the dynamometer footplate during active and passive trials.

Electrical activity (EMG) of the medial gastrocnemius (MG), soleus (Sol), and tibialis anterior (TA) muscles were recorded using Trigno Wireless electrode sensors (Delsys Inc, Ltd., Boston, USA). The EMG system had a predetermined bandwidth filter of 20‐450 Hz, gain of 1000, and was sampled at 2000 Hz. The EMG and dynamometry signals (joint torque, joint angle, and angular velocity) were synchronously collected with a data acquisition system, analog‐to‐digital converter (CED 1401, CED Cambridge, UK) and stored on a laptop using Spike 2 v7.21 software (CED, Cambridge, UK) at a sampling rate of 2 kHz for off‐line analysis.

A seven‐camera 3D motion analysis system (MAC Eagle, Motion Analysis Corporation Inc, Santa Rosa, CA., USA) interfaced with Cortex 1.0 software (Motion Analysis Corporation Inc, Santa Rosa, CA., USA) tracked the position of 12‐mm retro‐reflective markers during the trials at 120 Hz. Markers were placed on the lateral aspect of the head of the 5th metatarsal bone, 1st metatarsal of the foot, lateral and medial malleoli, calcaneal tuberosity, medial and lateral epicondyles of the femur of the dominant leg of the participants. Ankle joint angle was monitored during passive rotation using foot (markers on the toes and calcaneal tuberosity) and shank (markers on the malleoli and femur) segments to provide an estimate of the length of the gastrocnemius MTU following mathematical modeling suggested by Grieve et al.^26^ To this end, MG fascicle length at rest in the neutral position (0° ankle joint angle), and its elongation at end RoM passive dorsiflexion was estimated using B‐mode ultrasonography (Echoblaster 128, UAB “Telemed”, Vilnius, Lithuania). A layer of water‐based gel (Henleys Medical Supplies Ltd., Hertfordshire, UK) was applied between the ultrasound probe and skin for enhanced acoustic transmission without depressing the dermal surface. The probe was aligned to the midline of the muscle so that it was approximately in the same plane as the muscle fascicles. The probe was fixed in position using a custom‐made holder and was securely bandaged to the leg with Cohesive Bandage (CURRAGH Veterinary Supplies, Culworth, Oxfordshire) which permitted a constant pressure caused by the probe to the dermal surface. An echo‐sensitive wire pasted over the skin was placed between the probe and the skin for reference. This marker was used to check for a constant probe position. An analog signal from the ultrasound system was used to synchronize ultrasonography with the motion data.

### 2.4 Procedures

Two examiners, who remained consistent in their roles, were involved in data collection. Each participant completed a 5‐minute standardized warm‐up on a cycle ergometer (Ergomedic 874E Monark, Stockholm, Sweden) at 90 W (for males) or 60 W (for females). Electrodes for EMG were placed on the MG, Sol, and TA following the procedure described by Herda et al.^2^ To assess flexibility, the maximum joint ankle angle was measured during a passive stretch of the triceps surae muscles to the point of discomfort. While strapped to the footplate of the dynamometry system, the participant's foot and ankle were manually rotated once to the maximum dorsiflexion angle. A slow angular velocity (≤5°/s) was used during the manual rotation pre‐ and post‐intervention and between participants by the same experimenter for consistency and to ensure that the stretch did not elicit any phasic reflex‐mediated muscle activity which was monitored using electromyography.^27^ Moreover, at this low angular velocities, viscoelastic properties of the musculotendinous tissue minimally affect tissue strain.^28^ We subjectively decided that RMS EMG (measured over 2 seconds) at end RoM passive dorsiflexion was considered as being negligible if its magnitude was ≤10% of the RMS EMG during active isometric plantarflexion (below). Throughout the movement, we encouraged the participant to relax and not resist the passive motion of the footplate. In relaxed participants, magnitude of EMG increases with dorsiflexion beyond 10°,^27^ and inevitably, there is a possibility of increased (involuntary) tonic muscle activity with the joint being held at the extreme position.

Approximately 1 minute later, the participants performed a ramped isometric plantarflexor contraction with the ankle and knee at zero degrees. We instructed participants to keep their heel on the plate during the contraction, gradually (over ~2 seconds) reach to the maximum voluntary isometric contraction (MVIC) and maintain it for a further 1‐2 seconds. Verbal encouragement was provided to the participant who could see a real‐time graph of the joint moment. The maximum RMS EMG (for MG, Sol, and TA) during isometric plantarflexion was calculated for each participant over 2 seconds around the peak value and used for the assessment of reflex contraction (above). The isokinetic dynamometry test, which was delivered after another minute of rest, included assessment of the peak concentric/eccentric torque of the ankle plantarflexors with a CON/ECC method. In this method, five cycles of concentric/eccentric contractions were performed at a velocity of 30°/s. The concentric and eccentric torque at neutral ankle position was recorded for each of the five trials, and the peak value was used in the subsequent statistical analysis. For the concentric/eccentric cycle, the participant was encouraged to push/resist as hard as possible and to complete the full range of motion.

Blindfolded participants were required to reproduce a prescribed “target force” four times. Target force was set at 50% of their MVIC at neutral ankle angle produced during (pre‐ to post‐stretching) measurement. For this task, we asked participants to produce force to their “perceived target force”. The aim was to match the “target force” as closely as possible. Participants received no verbal or visual feedback from the test administrator to improve performance precision. In this way, participants were blinded to both the absolute level of the prescribed target force and the magnitude of measurements. Four trials were recorded. A rest period of 10 seconds was introduced between trials. A target force and lower scores reflect better sensorimotor performance. Force error was calculated using the equation: FE = (|(observed force – target force)|/target force) × 100%, and mean force error of the four trials was used for subsequent data analysis.

A test of positional error (PE) concluded pre‐stretching stage and involved evaluating a participant's ability to reproduce specific joint angles.^29^ Reproduction of the ankle angle was performed in a “closed kinetic chain” manner, with the participant position as described previously. From a “reference” ankle angle (20° plantarflexion), the experimenter passively moved the participant's ankle to the “target” neutral ankle angle (0°) and held this “target” position for 3 seconds and then returned it to the “reference” angle. The experimenter then moved the foot platform in the dorsiflexion direction again until the participant verbally indicated positional congruence of the ankle joint with the “target” angle while blindfolded in three discrete trials. The mean angular error associated with the three trials was recorded. The level of sensorimotor performance was accordingly calculated using the following equation PE = |(reproduced angle – target angle)|/number of trials.

### 2.5 Interventions

**Figure 3:**
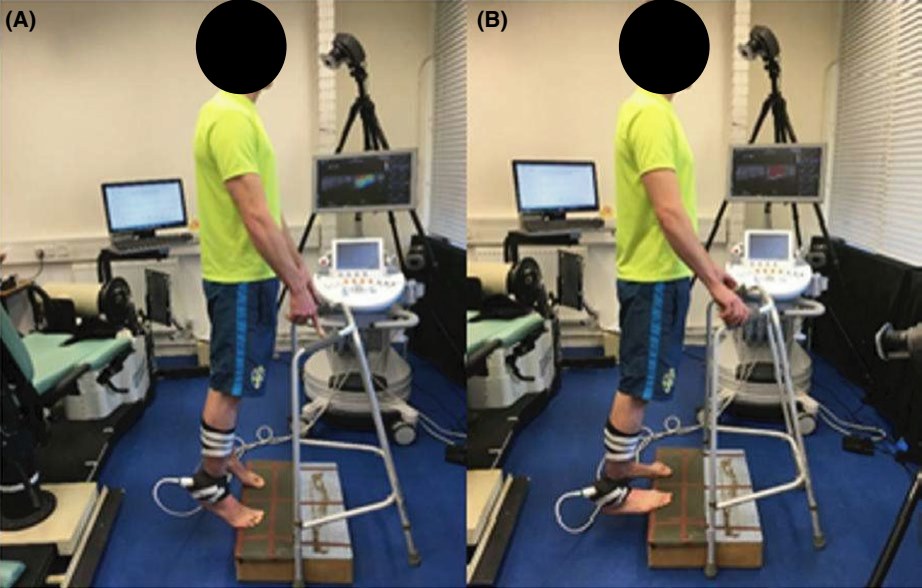
A. Start and finish position. Standing erect on the step. B. Position at full stretch

Dynamic stretching by definition consists of controlled, rhythmic, repeated movements through the active range of motion to the point of tension and return to full inner position incorporating sports‐specific movements which prepares the athlete's body for activity.^8^ Dynamic stretching started from a standing position off the edge of a wooden step with the heels raised (Figure 3). The participants balanced on the balls of their feet and held on loosely to a walker for stability. Then, they lowered and raised the heels in coordination with the beats of a metronome (MetroTimer 3.3.2, ONYX 3 Apps, Sofia, Bulgaria) either 50 or 100 beats/min three sets of 20 repetitions with a 5‐second rest in between each set. Medial gastrocnemius was the target muscle for the stretching protocol since during the stretching active plantarflexion (concentric contraction of MG), and dorsiflexion (eccentric contraction of the MG) ensured contraction of the “agonist” muscle group (ankle plantarflexors). Participants were instructed to move into full plantarflexion and dorsiflexion during the protocol.

### 2.6 Data processing

Torque (dynamometry) data were smoothed using a 200‐data point moving average window. Kinematic data used for determination of MTU length were filtered using a low‐pass, fourth‐order, zero‐lag Butterworth filter with a cutoff frequency of 6 Hz. Filtered motion analysis and dynamometry data were down‐sampled to 20 Hz to match the sampling frequency of the ultrasound data. The ultrasound data were then synchronized with kinematic and dynamometry data by using the cine loop synchronization output of the ultrasound system and using a Matlab script to export ultrasound frames with time stamps to match the kinematic and dynamometry data frames.

**Figure 4:**
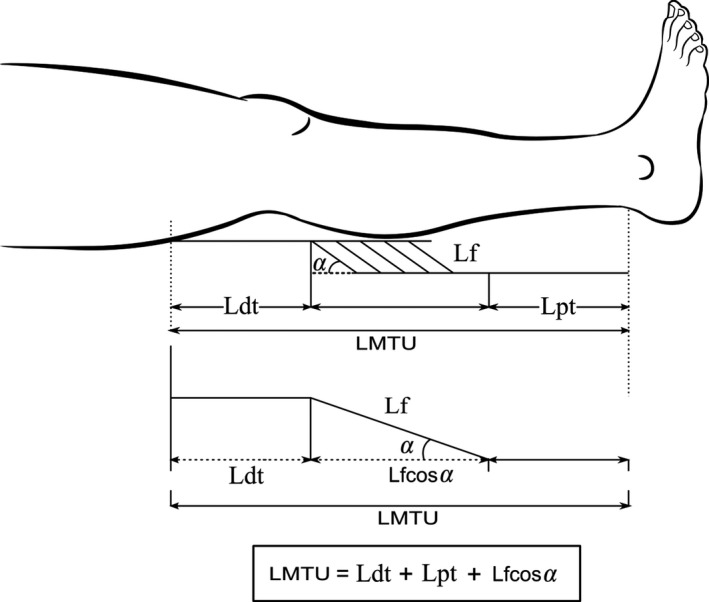
The musculotendon model used to estimate tendon length changes. Lf is the fascicular length, *α* is the pennation angle, Lpt is the proximal tendon (free tendon and aponeurosis) length, Ldt is the distal tendon (free tendon and aponeurosis) length, and Lmtu is the musculotendon unit length. The total tendon length (Lpt +Ldt) equals Lmtu‐Lfcos*α*

Ultrasound images were digitized using custom‐written routines in Matlab (MathWorks, Inc; Natick, MA). Pennation angle was measured as the angle between the fascicular path and the deep aponeurosis. Fascicle length (l_f_) was estimated using muscle thickness (*t*)*,* defined as the perpendicular distance between the superficial and deep aponeuroses, and the pennation angle (θ) according to l_f_ = t/sin θ^30^ (Figure 4).

Ultrasound images were taken at approximately the mid‐belly of the muscle as changes at this site have been shown to be relatively uniform.^31^ We identified and selected three optimal and identifiable fascicles together with the deep and superficial aponeuroses. These fascicles were tracked in each frame of the pre‐ and post‐stretch trials, and an average of the three fascicles was used for subsequent analysis. The pennation angle was determined from the mid‐belly of the medial gastrocnemius muscle. Muscle, tendon, and MTU lengths were calculated from a combination of motion analysis and ultrasound data. The MTU (2D) length at each joint angle was estimated using a cadaveric regression model,^26^ while muscle and tendon lengths were calculated after accounting for the changes in the pennation angles and muscle fascicles according to the musculotendon model by Fukunaga et al^32^ Strain of the MG muscle and tendon was calculated as the percentage of the change in length to the resting length.

### 2.7 Statistical analysis

Data are presented as mean ± standard deviation (SD). Within‐group changes were analyzed using a post‐only crossover trial with adjustment for a predictor (covariance) spreadsheet,^33^ and between‐group changes were analyzed using a pre‐post parallel groups trial spreadsheet.^34^ This pre‐post parallel group spreadsheet also calculated individual differences in response to an intervention, which are often highly variable.^34^ Differences between trials were expressed as percentages determined from log‐transformed and subsequently back‐transformed data, with 90% confidence intervals (CI) reported as estimates of uncertainty to quantify the magnitude of the difference between pre‐intervention and post‐intervention outcome performance measures.^35^ According to Hopkins et al,^35^ this is the appropriate method for quantifying changes in athletic performance. Dependent variables were analyzed either as log‐transformed data (Torque, Muscle strain, Tendon strain, Passive torque, FE, PE) or raw data (RoM, Pennation angle).^36^ In athletic performance research, it has been argued that it is not whether an effect exists but how big the effect is that matters, and the use of the *P*‐value alone provides no information about the direction or size of the effect or the range of feasible values.^35^ The magnitude of the effect size was classified as trivial (<0.2), small (0.2‐0.6), moderate (0.6‐1.2) or large (2.0‐4.0), and extremely large (>4.0) via standardized thresholds.^35^ The threshold value for the smallest worthwhile change was set at 0.2 between‐subject standard deviation. Mechanistic inference was then based on the disposition of the 90% confidence interval for the mean difference to this smallest worthwhile effect; the probability (percent chances) that the true population difference between trials is substantial (beneficial/detrimental) or trivial was calculated as per the magnitude‐based inference approach.^37^ Where the 90% CI overlapped the thresholds for the smallest worthwhile change in both positive and negative sense, the true effect was classified as unclear. In the event that a clear interpretation was possible these percent chances were qualified via probabilistic terms assigned using the following scale: <0.5%, most unlikely or almost certainly not; 0.5%‐5%, very unlikely; 5%‐25%, unlikely or probably not; 25%‐75%, possibly; 75%‐95%, likely or probably; 95%‐99.5%, very likely; and >99.5%, most likely or almost certainly.^35^

## 3 RESULTS

Tables 1 and 2 show pre‐ and post‐DS values with mean differences, effect sizes, and qualitative non‐clinical inferences based on post‐only crossover trial analysis. Table 3 presents the standardized difference changes in the data (mean difference ± 90% CI) and qualitative non‐clinical inferences between stretching treatments based on pre‐post‐only crossover trial analysis.

**Table 1:** Descriptive statistics and mean differences in the SDS performance measures along with effect sizes and qualitative inferences

Performance measures	Pre‐stretching (mean ± SD)	Post‐stretching (mean ± SD)	Mean differences (±90% CI)	Effect sizes (±90% CI)	Likelihood (%) of SDS being increase/trivial/decrease	Qualitative inferences
Maximum passive ankle dorsiflexion RoM (°)	19.26 ± 6.34	21.04 ± 7.85	1.80 ± 1.20	0.27 ± 0.18	75/25/0	Likely increase
Peak isometric torque (Nm)	91.10 ± 26.59	95.32 ± 26.07	5.20 ± 3.50	0.15 ± 0.10	19/81/0	Likely trivial
Peak concentric torque (Nm)	77.56 ± 27.39	84.74 ± 22.94	14.10 ± 11.80	0.26 ± 0.28	70/30/0	Possibly increase
Peak eccentric torque (Nm)	83.89 ± 28.16	90.80 ± 22.06	11.40 ± 9.00	0.24 ± 0.18	65/35/0	Possibly increase
Passive peak torque (Nm)	20.26 ± 6.60	23.4 ± 9.00	13.90 ± 8.20	0.35 ± 0.19	90/10/0	Likely increase
Muscle strain (%)	20.10 ± 13.78	13.03 ± 9.67	−38.00 ± 19.70	0.66 ± 0.43	0/4/96	Very likely decrease
Tendon strain (%)	1.00 ± 2.06	2.33 ± 1.41	49.50 ± 35.20	0.77 ± 0.45	98/2/0	Very likely increase
MTU strain (%)	3.23 ± 1.00	3.50 ± 1.23	5.80 ± 6.90	0.16 ± 0.18	34/65/0	Possibly increase
Resting pennation angle (°) at neutral	15.7 ± 2.84	16.00 ± 3.09	0.30 ± 0.60	0.10 ± 0.20	20/79/1	Likely trivial
Force error (%)	20.63 ± 12.30	20.82 ± 12.28	−3.6 ± 36.50	0.05 ± 0.53	21/47/32	Unclear get more data
Positional error (°)	3.15 ± 2.58	2.50 ± 3.21	−24.1 ± 35.00	0.24 ± 0.39	3/40/57	Possibly decrease

Torque, Muscle Strain, Tendon Strain, Passive Torque, Force Error, and Positional Error are reported as log‐transformed data. RoM and Pennation Angle are reported as raw data.

**Table 2:** Descriptive statistics and mean differences in the FDS performance measures along with effect sizes and qualitative inferences

Performance measures	Pre‐stretching (mean ± SD)	Post‐stretching (mean ± SD)	Mean differences (±90% CI)	Effect sizes (±90% CI)	Likelihood (%) of SDS being increase/trivial/decrease	Qualitative inferences
Maximum passive ankle dorsiflexion RoM (°)	18.97 ± 6.22	21.07 ± 6.28	2.10 ± 0.80	0.32 ± 0.19	86/14/0	Likely increase
Peak isometric torque (Nm)	93.85 ± 26.07	96.86 ± 28.28	3.80 ± 3.60	0.10 ± 0.10	5/95/0	Likely trivial
Peak concentric torque (Nm)	84.74 ± 22.94	90.71 ± 28.60	8.00 ± 6.40	0.18 ± 0.14	40/60/0	Possibly increase
Peak eccentric torque (Nm)	90.80 ± 27.06	95.84 ± 20.69	5.30 ± 4.10	0.12 ± 0.09	8/92/0	Unlikely increase
Passive peak torque (Nm)	20.96 ± 5.54	23.52 ± 7.61	10.50 ± 8.50	0.35 ± 0.27	83/17/0	Likely increase
Muscle strain (%)	24.67 ± 12.92	21.17 ± 13.02	−13.20 ± 20.40	0.23 ± 0.36	3/42/55	Possibly decrease
Tendon strain (%)	−0.81 ± 1.17	0.94 ± 1.53	41.40 ± 44.90	0.73 ± 0.66	92/6/2	Likely increase
MTU strain (%)	3.20 ± 1.02	3.48 ± 0.99	10.20 ± 7.40	0.28 ± 0.19	76/24/0	Likely increase
Resting pennation angle (°) at neutral	15.37 ± 3.13	15.25 ± 3.13	−0.60 ± 1.00	0.17 ± 0.30	2/53/44	Likely trivial
Force error (%)	16.06 ± 11.61	20.18 ± 13.78	31.40 ± 82.90	0.19 ± 0.42	49/45/6	Unclear get more data
Positional error (°)	2.44 ± 2.90	+2.78 ± 2.76	20.80 ± 58.10	0.16 ± 0. 38	42/52/6	Unclear get more data

Torque, Muscle Strain, Tendon Strain, Passive Torque, Force Error, and Positional Error are reported as log‐transformed data. RoM and Pennation Angle are reported as raw data.

**Table 3:** Descriptive statistics and mean differences, for the between‐group comparisons along with effect sizes and qualitative inferences

Performance measures	Differences between groups (FDS‐SDS) (Mean difference; ±90% CI)	Effect sizes (±90% CI)	Likelihood (%) of SDS being increase/trivial/decrease	Qualitative inferences
Maximum passive ankle dorsiflexion (°)	0.30 ± 1.50	0.05 ± 0.24	15/80/5	Likely trivial
Passive peak torque (Nm)	−2.70 ± 8.40	0.09 ± 0.27	4//72/24	Unlikely decrease
Peak isometric torque (Nm)	−1.20 ± 4.30	0.04 ± 0.13	0/98/2	Very likely trivial
Peak concentric torque (Nm)	−4.80 ± 13.30	0.11 ± 0.31	5/64/31	Possibly decrease
Peak eccentric torque (Nm)	−4.90 ± 11.00	0.12 ± 0.19	3/67/31	Possibly decrease
Muscle strain (%)	39.80 ± 56.70	0.50 ± 0.89	80/17/3	Likely increase
Tendon strain (%)	13.20 ± 20.10	0.30 ± 0.49	5/32/64	Possibly decrease
MTU strain (%)	4.60 ± 10.60	0.13 ± 0.29	34/62/3	Possibly increase
Resting pennation angle (°) at neutral	−0.40 ± 0.90	0.15 ± 0.28	2/60/38	Possibly decrease
Force error (%)	80.10 ± 143.50	0.40 ± 0.52	74/23/3	Possibly increase
Positional error (°)	54.90 ± 87.90	0.38 ± 0.47	74/24/2	Possibly decrease

Torque, Muscle Strain, Tendon Strain, Passive Torque, Force Error, and Positional Error are reported as log‐transformed data. RoM and Pennation Angle are reported as raw data.

### 3.1 Ankle flexibility and strength

Maximum passive ankle dorsiflexion at end RoM showed a *possible* improvement after the SDS (mean difference ± 90% CI; 1.8 ± 1.2°; small effect) and a *likely* improvement after the FDS (2.1 ± 1.2°; small effect). For changes between SDS and FDS treatment, there was a *likely trivial* effect in the maximum passive ankle dorsiflexion RoM when comparing SDS and FDS treatments (0.05 ± 0.24°, trivial effect).

Slow dynamic stretching (5.2 ± 3.5%; trivial effect) and FDS (3.8 ± 3.6%; trivial effect) resulted in a *likely trivial* increase in peak plantarflexor isometric torque. For changes between SDS and FDS treatment, there was a *very likely trivial* effect on peak plantarflexor isometric torque (−0.04 ± 0.13%; trivial effect).

Peak plantarflexor concentric torque at neutral ankle position demonstrated a *possible* increase after both SDS (14.1 ± 11.8%; small effect) and FDS (8.0 ± 6.4%; small effect). Comparing the two treatment protocols for changes between the SDS and FDS protocols, the SDS protocol resulted in a *possible* greater improvement compared to FDS (−4.8 ± 13.3%; trivial effect).

Slow dynamic stretching resulted in a *possible* increase in peak plantarflexor eccentric torque (11.4 ± 9.0%; small effect), but FDS resulted in an *unlikely* increase on peak plantarflexor eccentric torque (5.3 ± 4.1%; trivial effect). For changes between SDS and FDS, there was a *possible* improvement in peak plantarflexor eccentric torque after SDS (−4.9 ± 11.0%; trivial effect).

### 3.2 Mechanical and architectural properties of the ankle joint, muscle, and tendon

Both protocols showed a *likely* increase on peak passive dorsiflexor torque at end RoM: (13.9 ± 8.2%; small effect) after the SDS protocol and (10.5 ± 8.5%; small effect) after the FDS protocol. For changes between the two protocols, there was an *unlikely* decrease (−2.7 ± 8.4%; trivial effect) after FDS in comparison to SDS.

Slow dynamic stretching (49.5 ± 35.2%; moderate effect) resulted in a *very likely*, and FDS (41.4 ± 44.9%; moderate effect) resulted in a *likely* increase in passive tendon strain. For changes between SDS and FDS, there was a *possible* greater decrease in passive tendon strain after FDS compared to SDS (13.2 ± 21.3%; small effect).

The SDS protocol resulted in a *very likely* decrease (−38.0 ± 19.7%; moderate effect), and the FDS protocol resulted in a *possible* decrease (−13.6 ± 20.4%; small effect) in passive muscle strain. For changes between SDS and FDS, there was a *likely* greater increase in passive muscle strain (39.8 ± 56.7%; small effect) after FDS compared to SDS.

Both SDS and FDS interventions resulted in a *possible* increase in passive MTU strain at end RoM: (5.8 ± 6.9%; trivial effect) for the SDS and (0.28 ± 0.19%; small effect) for FDS, respectively. For changes between SDS and FDS, there was a *possible* greater increase in passive MTU strain (0.13 ± 0.29%; trivial effect) after FDS in comparison to SDS.

Both SDS and FDS protocols resulted in a *likely* trivial change in pennation angle at neutral position: (0.3 ± 0.6°; trivial effect) for SDS and (−0.6 ± 1.0°; trivial effect) for FDS, respectively. For changes between the SDS and the FDS protocol, there was a *possible* decrease on the pennation angle at neutral position after SDS compared to FDS (−0.4 ± 0.9°; trivial effect).

### 3.3 Sensorimotor performance

There were no clear effects of DS protocols on FE (−3.6 ± 36.5%; trivial effect for the SDS, and 31.4 ± 82.9%; trivial effect for the FDS). For changes between the SDS and FDS on FE, there was a *possible* increase (8.0 ± 149.5%; small effect) after FDS in comparison to SDS. On the PE, SDS protocol resulted in a *possible* better performance in achieving “target” angle (−24.0 ± 35.0%; small effect), but the effect of FDS on PE was *unclear* (20.8 ± 54.1%; trivial effect). Comparing the two protocols, there was a *possible* increase in PE score after FDS compared to SDS (54.9 ± 87.9%; small effect).

## 4 DISCUSSION

The purpose of our study was to determine whether the expected increase in passive RoM of the ankle joint in response to DS would be accompanied by changes in the MG muscle and tendon mechanical behavior, plantarflexor torque, and ankle joint PE and FE acuity. Additionally, we wanted to investigate possible differences in the effects of SDS and FDS on these neuromechanical and sensorimotor performance parameters. As we anticipated, both DS protocols increased flexibility at the ankle joint. Neither of the protocols showed any superiority in increasing joint flexibility over the other. The change in the end RoM after acute bouts of DS was due to a relatively larger increase in the strain of the tendon than the muscle, and there was a possible difference between the two protocols with FDS *possibly* resulting in a greater decrease in tendon strain compared to SDS. There was no stretch‐induced impairment in peak isometric torque following both acute DS protocols, and the effects of DS protocols on the maximum concentric and eccentric torques were not detrimental. SDS showed greater superiority in isotonic strength performance compared to FDS. Results were unclear regarding the effect of DS protocols on the FE and PE except for the SDS which showed *possibly *beneficial effects post‐intervention.

Improvement in flexibility after DS was in agreement with previous studies that used similar DS protocols (contracting the muscle group “agonist” to the target muscle group), as was performed in this study.^13^, ^14^ After both protocols, MTU strain was larger, but there was a decrease in muscle strain during passive dorsiflexion compared to the pre‐stretching stage, and accordingly, muscle contribution to the increase in the overall elongation of the MTU was decreased. These findings are in agreement with a previous study. Samukawa et al^14^ found no change in the muscle fascicle length and/or decrease in the pennation angle (suggesting an increase in tendon length) after DS in standing participants. However, the ankle and knee angles were not controlled pre‐ and post‐DS testing in that study to ensure consistency of posture. Recently, Pamboris et al^38^ found a decrease in muscle fascicle strain combined with an increase in muscle stiffness (measured by shear wave elastography) attributing the increase to ankle dorsiflexion RoM to increased tendon strain. Mizuno and Umemura^16^ reported an increase in maximum passive ankle dorsiflexion RoM without changing the passive stiffness of the MTU attributing it to an increase in pain tolerance. Our results cannot refute that alteration in pain tolerance (ie, discomfort or pain perception at a given ROM) is a contributing mechanism to the increased RoM changes after stretching.

The effect of other protocols, resembling DS as employed in the present study, suggests a reduction in tendon stiffness in response to the intervention. Kubo et al^11^ reported that 50 repetitions of 3 seconds MVIC of the MG decreased its tendon stiffness, while Maganaris et al^39^ found that 10 repeated MVIC plantarflexion contractions at 80% of the peak moment resulted in a decrease in fascicle length, increase in pennation angle, and myotendinous junction displacement. Direct stimulation is considered necessary to change the mechanical behavior of the MTU.^11^, ^39^ Thus, studies in which DS involved “agonist” muscle group contractions demonstrated decreased in mechanical behavior,^13^, ^14^ while in Mizuno and Umemura,^16^ whose DS protocol involved “antagonist” muscle group contractions, there was no change in MTU behavior.

Despite different contributions from tendon and muscle in response to different protocols, flexibility did not differ between the two conditions (Table 3). Comparing the two interventions, we can conclude that muscle strain increases and tendon strain decreases as the velocity of DS intervention increases from SDS to FDS. The present results suggest that stretching parameters such as the velocity could differentially affect mechanical characteristics of the muscle and tendon. Overall, the increased strain of tendon in response to the acute bouts of DS might have important implications during functional activities via altering length‐tension properties of the MTU,^40^ muscle fascicle shortening velocity, tendon's elastic storage capacity,^41^, ^42^ and rate of force development.^43^

The results of this study showed no stretch‐induced impairment in peak plantarflexor torque following the two acute DS protocols. This finding is consistent with other studies that found DS did not have a detrimental effect on the isometric strength of the leg flexors^2^; neither protocol showed any superiority over the other. At least two mechanisms can be involved in preserving strength after stretching: First, we can speculate a potentiation of the subsequent performance during the 2‐minute recovery period for isometric contraction. Second, it has been suggested that with smaller pennation angles, the muscle has a mechanical advantage for force transmission to the tendon.^44^ Measuring pennation angle immediately after SDS and FDS, we found no change from pre‐stretching values.

The effects of both DS protocols on the maximum concentric and eccentric torques were certainly not detrimental. These findings contradict a report that found a significant decrease in knee flexors concentric and eccentric torques at speeds of 60°/s and 180°/s.^45^ On the contrary, other studies have found a significant increase in the concentric and eccentric peak torque of the hamstrings and the quadriceps at 60°/s and 180°/s.^4^, ^46^ The discrepancy between these and our results might be attributed to methodological issues such as the employment of different stretching/experimental protocols (intensity, volume, duration of rest intervals between the consecutive sets, velocity of isokinetic assessments), gender,^47^ muscle groups, and time interval (ranging 2‐5 minutes) between completion of the intervention and assessment. Comparing the two interventions, the SDS showed a *possibly* greater increase in concentric and eccentric strength performance compared to FDS. This is quite surprising since the participants studied by Fletcher^17^ showed an increase in jump performance in FDS compared to FDS. This might be explained by the different outcome measures used in our study. It has been suggested that muscles work optimally when their frequency of force application (ie, movement frequency) matches the system's natural frequency^48^ to cause resonance (greatest oscillation amplitude for least input effort). Additionally, based on the current results, our findings are also probably explained by the principle of training specificity. Although not measured directly, the velocity of SDS might have been more similar to the velocity of muscle contraction during isokinetic tasks (30°/s), which possibly benefited torque production during dynamic movements.

The findings of our study were unclear regarding the effect of DS protocols on the force matching task. On the PE test, an acute bout of SDS had a small positive effect while the effect of FDS was unclear. Despite offering no clear conclusions based on the present findings, we suggest that conformations in MTU and tendon might affect the function of the proprioceptors such as tendon organs together with muscle spindle fibers^49^ with implications for risk of musculoskeletal injury, capabilities of the musculature to act synergistically, and dynamic stabilization of the joint system which warrant further examination.^29^

Although one of the main limitations in the present study is the non‐inclusion of a control group, these are standard techniques which are used in the literature without the inclusion of a control group^38^, ^50^, ^51^ and participants were randomly assigned into the two groups, SDS and FDS. Moreover, we instructed the participants to refrain from vigorous physical activity for 48 hours before the testing sessions in order to avoid any potential carryover effects and to promote neuromuscular recovery. In order to minimize the carryover effects of voluntary efforts on performance outcomes, assessments were sequenced within each testing day as follows: passive ankle flexibility, voluntary neuromuscular performance, and sensorimotor performance (see Figure 1).

## 5 PERSPECTIVES

Measures of strength were preserved and certainly not adversely affected by stretching and ankle joint flexibility (RoM) increased with both DS protocols. Results of the sensorimotor performance (FE and PE) should be considered with caution as they were mainly unclear. Therefore, one can conclude that performing either of the two DS protocols was not detrimental to performance. We suggest that incorporation of a DS protocol that closely matches the kinematics of the activity to be undertaken could be more likely to deliver benefits of stretching although when assessment of performance outcomes is carried out shortly (2 minutes) after DS. In the light of current findings, we can recommend DS in warm‐up routines but cannot comment on its effect on injury prevention.
